# Chronic Hypobaric Hypoxia Modulates Primary Cilia Differently in Adult and Fetal Ovine Kidneys

**DOI:** 10.3389/fphys.2017.00677

**Published:** 2017-09-20

**Authors:** Kiumars Shamloo, Juan Chen, Jasmine Sardar, Rinzhin T. Sherpa, Rajasekharreddy Pala, Kimberly F. Atkinson, William J. Pearce, Lubo Zhang, Surya M. Nauli

**Affiliations:** ^1^Department of Biomedical and Pharmaceutical Sciences, Chapman University Irvine, CA, United States; ^2^Departments of Basic Sciences, Physiology and Pharmacology, Lawrence D. Longo MD Center for Perinatal Biology, Loma Linda University School of Medicine Loma Linda, CA, United States; ^3^Division of Nephrology and Hypertension, Department of Medicine, University of California, Irvine Irvine, CA, United States

**Keywords:** cilium, kidney injury, mechanosensor, pO_2_, sheep, shear-stress, renal fibrosis

## Abstract

Hypoxic environments at high altitude have significant effects on kidney injury. Following injury, renal primary cilia display length alterations. Primary cilia are mechanosensory organelles that regulate tubular architecture. The effect of hypoxia on cilia length is still controversial in cultured cells, and no corresponding *in vivo* study exists. Using fetal and adult sheep, we here study the effect of chronic hypobaric hypoxia on the renal injury, intracellular calcium signaling and the relationship between cilia length and cilia function. Our results show that although long-term hypoxia induces renal fibrosis in both fetal and adult kidneys, fetal kidneys are more susceptible to hypoxia-induced renal injury. Unlike hypoxic adult kidneys, hypoxic fetal kidneys are characterized by interstitial edema, tubular disparition and atrophy. We also noted that there is an increase in the cilia length as well as an increase in the cilia function in the hypoxic fetal proximal and distal collecting epithelia. Hypoxia, however, has no significant effect on primary cilia in the adult kidneys. Increased cilia length is also associated with greater flow-induced intracellular calcium signaling in renal epithelial cells from hypoxic fetuses. Our studies suggest that while hypoxia causes renal fibrosis in both adult and fetal kidneys, hypoxia-induced alteration in cilia length and function are specific to more severe renal injuries in fetal hypoxic kidneys.

## Introduction

Hypoxic environments in humans can result in the high-altitude renal syndrome (HARS) (Arestegui et al., 2011). HARS is characterized by reduced renal plasma flow (Becker et al., 1957), microalbuminuria (Becker et al., 1957), proteinuria (Jefferson et al., 2002; Chen et al., 2011), hyperuricemia (Jefferson et al., 2002) and systemic hypertension (Jefferson et al., 2002; Gilbert-Kawai et al., 2016). The kidneys have a fundamental role in adaption to regulate body fluids, electrolyte and acid-base homeostasis during acclimatization to high altitude and in mountain sickness syndromes. Kidneys also respond to hypoxic diuresis and natriuresis through inhibition of renal tubular sodium reabsorption, in addition to erythropoietin production (Haditsch et al., 2015).

Kidneys are very susceptible to lowered oxygen tensions (Mayer, 2011; Fu et al., 2016). In acute hypoxia, kidneys exhibit defense mechanisms centered around the activation of hypoxia-inducible factor (HIF) (Minet et al., 2000; Rosenberger et al., 2006). HIF up-regulates pro-angiogenic factors that protect against capillary rarefaction. HIF also increases expression of matrix metalloproteinases that effect repair and protect against fibrosis by degrading extracellular matrix. In chronic hypoxia, however, HIF mRNA is destabilized, shutting-down the kidney's initial protective mechanisms (Uchida et al., 2004). The expression of pro-angiogenic factors is repressed, and the expression of dys-angiogenic factors is increased. This may thus result in capillary rarefaction and potential fibrosis.

Primary cilia are sensory organelles that sense extracellular milieu along the tubule of a nephron. Abnormal cilia length and function result in polycystic kidneys (Nauli et al., 2003, 2006; Xu et al., 2007). Renal cilia also display length and function alterations following kidney injury. Primary cilia play a crucial role in cisplatin-induced tubular apoptosis by regulating tubular apoptotic pathway (Wang et al., 2013). Primary cilia also undergo abnormal changes in renal transplant biopsies with acute tubular injury (Hayek et al., 2013). During injury, renal cilia decrease in length in both humans and mice (Verghese et al., 2008, 2011). The shortened cilia length serves to attenuate cilia-mediated signaling pathways in response to extracellular stress, which promotes infiltration of neutrophils and macrophages in the kidney (Prodromou et al., 2012). However, the effects of chronic hypobaric hypoxia on primary cilia have yet to be investigated.

Changes in fluid-shear stress generated by urine movement can also contribute to kidney injury if the primary cilia are not functioning normally. Thus, the change in urinary flow associated with nephropathies has been proposed to be a potential insult for tubular cells leading to disorganization of the tubular epithelium (Maggiorani et al., 2015).

Based on the evidence above, it has been proposed that cilia play important roles in sensing environmental cues caused by injury and in the repair process for reestablishing a new epithelial layer of differentiated cells (Bell et al., 2011; Wang and Dong, 2013). By using a series of human renal transplant biopsies, it was found that acute tubular necrosis is associated with more than two-fold longer cilia 1-week after kidney injury, and normalization of cilium length occurred at a later stage. These results indicate that cilia function could be a clinically relevant indicator of kidney injury and repair in patients with kidney transplantation (Wang and Dong, 2013).

Despite the fact that hypoxic environment at high altitude may have significant effects on the kidneys, the histological analysis of renal architecture has never been examined especially in the fetus. Given the background information, our premises indicate that hypoxia causes renal injury/damage and that renal injury often includes altered cilia structure/function. Thus, our hypothesis is that hypoxia causes altered cilia structure/function. Here, we examined the effects of hypoxic high-altitude on the kidneys in adult and fetal sheep. We also took this opportunity to study cilia length-function relationship in response to chronic hypobaric hypoxia, because it has been reported that HIF could alter the structural length of primary cilia in hypoxic *in vitro* models (Verghese et al., 2011; Ding et al., 2015; Lavagnino et al., 2016; Resnick, 2016).

## Methods

The sheep were purchased from Nebeker Ranch, Inc. (Lancaster, CA). The normoxic control animals were maintained at sea level throughout the gestation period (300 m). To induce chronic high-altitude hypoxia, the animals were then transported at 30 days of gestation to the Barcroft Laboratory, White Mountain Research Station at Bishop, CA (3,801 m). The hypoxic animals stayed in Bishop for 110 days. Both normoxic control and hypoxic animals were fed Alfalfa hay in a “keyhole” feeder in addition to providing a mineral block *ad libitum*. The animals were transported from Bishop to the laboratory immediately before the studies (Dasgupta et al., 2012; Thorpe et al., 2013).

Two-years-old pregnant sheep and near-term 146 gestational day fetal lambs were used. Please note that sheep gestation typically lasts ∼150 days. The sheep were anesthetized with thiamylal (10 mg/kg, i.v.) followed by inhalation of 1.5–2.0% halothane. An incision was made in the abdomen, and kidneys were isolated and immediately placed in either 10% buffer formalin or the phosphate-buffered sucrose solution (30% sucrose, 137 mM NaCl, 2.7 mM KCl, 10 mM Na_2_HPO_4_, 1.8 KH_2_PO_4_, 1 mM CaCl_2_•2H_2_O and 0.5 mM MgCl_2_•6H_2_O at pH 7.4). All procedures and protocols were conducted in full compliance with the Animal Welfare Act, followed the guidelines by the National Institutes of Health Guide for the Care and Use of Laboratory Animals, and were approved by the Institutional Animal Care and Use Committee at Loma Linda University, CA.

### Tissue processing

Kidney cross-sections of the cortex and medulla were dehydrated with isopropyl alcohol and infiltrated with paraffin in a tissue processor (Excelsior AS, Thermo Scientific, Inc.). Tissues were allowed to solidify in a base mold and then snapped into plastic tissue cassettes using a paraffin molding processor (HistoStar, Thermo Scientific, Inc.). Samples were cut to 5 μm thickness sections with a microtome (HM 355 S, Thermo Scientific, Inc.), and continuous “ribbon” of the sections were formed. Sections were carefully transferred onto a 40°C water dish for about 3–5 min to flatten and avoid wrinkles in the sectioned tissues.

Tissue slices were placed on the charged microscope slides, and deparaffinization was performed by placing the slides in a 60°C oven for 30 min in order to melt any extra paraffin. Slides were rinsed twice with a xylene solution for 5 min each, followed by hydration through a series of washing for 1 min each in decreasing alcohol concentrations (100, 95, 80, 70%). Slides were then submerged in the distilled water for 3 min for the staining process.

### Tissue staining

For H&E staining, tissues were stained with hematoxylin for 5 min, rinsed with tap water for 1 min and dipped for 1 sec in acid alcohol (200 ml of 50% alcohol containing 500 μL HCl 5N). Tissues were then stained with Eosin for 2 min and rinsed with distilled water for 1 min followed by dehydration with 95% and 100% alcohol each for 1 min and cleaning with xylene for 2 min. Coverslip was mounted onto a slide with xylene compatible mounting media.

For periodic acid Schiffs (PAS) staining, staining kit (*Polysciences, Inc*.) was used and experiments were performed based on the Company's protocol. Briefly, tissues were oxidized in 0.5% periodic acid for 5 min. After washing with deionized (DI) water, tissue sections were placed in Schiff's reagent for 15 min then rinsed with 0.55% potassium metabisulfite. Slides were washed in tap water for 10 min to allow the color to develop in the tissues, which were then counterstained with acidified Harris Hematoxylin for 30 s. Slides were washed with tap water until the tissues turned to a blue color. After dehydration with 95 and 100% alcohol each for 1 min and clearing with xylene for 2 min, the coverslips were mounted onto the slides with a xylene compatible mounting media.

For Masson's Trichrome staining, a staining kit (*Polysciences, Inc*.) was used according to the manufacture's instructions. Briefly, tissues were incubated in Bouin's fixative solution at 60°C for 1 h and rinsed with warm tap water for 2–3 min until the yellow color disappeared. Fresh Weigert's iron hematoxylin working solution was prepared by mixing Weigert's hematoxylin A and Weigert's hematoxylin B in a 1:1 ratio. Tissues were incubated in the working solution for 20 min and rinsed for 5 min with distilled water. Tissues were then stained with Biebrich Scarlet-Acid Fuchsin solution for 15 min and rinsed with tap water to remove any extra solution-residue. After rinsing with distilled water, the tissues were soaked in Phosphotungstic / Phosphomolibdic acid for 10 min, transferred to Aniline Blue for 7 min and washed with distilled water until the slides were clear. Tissues were then soaked in 1% acetic acid for 1 min and washed with distilled water for 1 min. After dehydration with 95 and 100% alcohol each for 1 min followed by cleaning with xylene for 2 min, the coverslips were mounted onto the slides with a xylene compatible mounting media.

For immunofluorescence staining, heat-induced epitope retrieval was performed using a pressure cooker and Tris-EDTA buffer (10 mM Tris base, 1 mM EDTA solution, 0.05% Tween 20, pH 9.0). Tris-EDTA buffer was first boiled in the pressure cooker to 100°C. Tissues were then maintained in the pressure cooker at about 121°C and 15 psi for 8 min. The pressure cooker and slides were then chilled quickly with cold tap water. The slides were rinsed three times with phosphate buffer saline (PBS) and blocked in PBS solution containing 5% bovine serum albumin for 15 min in a humidifying chamber. Using our standard protocol (Loghman-Adham et al., 2003; Nauli et al., 2003, 2006), tissues were stained with the ciliary marker acetylated-α-tubulin (1:1,000 dilution; Sigma-Aldrich, Inc.), distal tubular marker bolichos biflorus agglutinin (DBA; 4:1,000 dilution; Vector Laboratory, Inc.), proximal tubular marker lotus tetragonolobusl lectin (LTL; 4:1,000 dilution; Vector Laboratory, Inc.), and nucleus marker DAPI (Vector Laboratory, Inc.).

### Primary cell culture

Isolation of renal epithelia from fresh tissues has been previously described in detail (Loghman-Adham et al., 2003; Nauli et al., 2003, 2006). After extensive washing in phosphate-buffered sucrose solution, the cortex and medullary regions of kidney were dissected into pieces and incubated with collagenase followed by 0.05% trypsin/0.53 mM EDTA at 37°C for 15–20 min. After vortexing vigorously for 5 min, ice-cold HBSS containing 10% FBS was added to inactivate the trypsin. The cells were further released from the fibrous basement membrane by trituration, washed twice with HBSS. Cells were then sorted based on their surface markers for DBA or LTL and resuspended in fresh culture medium. To allow attachment, the cells was grown in DMEM containing 10% FBS and supplemented with 5 μg/ml insulin, 5 μg/ml transferrin, 5 ng/ml selenium, 36 ng/ml (10^−7^ M) hydrocortisone, 10^−8^ M triiodothyronine, 10 ng/ml EGF, and 50 ng/ml PGE1, as well as 100 U/ml penicillin, and 100 μg/ml streptomycin in a 37°C humidified incubator ventilated with 5% CO_2_—95% O_2_. The culture medium was changed every 2–3 days until confluency was reached.

### Cilia function and length measurements

Bending primary cilia with fluid-flow activates the cilium and increases cytoplasmic calcium in renal epithelial cells (Praetorius and Spring, 2001; Liu et al., 2005; Jin et al., 2014). Thus, intracellular calcium was measured with Fura2-AM [to study cilia function] as previously described (Nauli et al., 2013). Briefly, after pre-incubation with 5 μM Fura2-AM for 30 min at 37°C, the tissues were equilibrated for at least a minute. The optimal shear-stress of 0.8 dyne/cm^2^ was used to monitor changes in cytosolic calcium as previously described (Nauli et al., 2013). Ionomycin (1 μM) was used at the end of each experiment to confirm Fura-2 loading and determine minimal and maximal ratiometric fluorescence signals. Cells were then analyzed for cilia length by staining with the ciliary marker acetylated-α-tubulin by staining with the ciliary marker acetylated-α-tubulin as previously described (Loghman-Adham et al., 2003; Nauli et al., 2003, 2006).

### Data analysis

All images represent the extracted information from the digital pictures. Images were captured through a Nikon Ti-E microscope. The Nikon NIS Elements for Advanced Research software was used for image capture and analysis, including automatic object recognition, image scanning and color binary segmentation. Scale bars were provided in all figures to indicate the actual image reduction size. All morphometric data were reported as mean ± SEM. Distribution analyses were performed and presented on all data sets to verify normal data distributions. After distribution and variance analyses, data comparisons for more than two groups were performed using ANOVA test followed by a Tukey *post-hoc* analysis. The correlation between cilia length and cilia function was analyzed with the ordinary least squares (OLS) regression of y on x, because the ordinary least products (OLP) would not allow analysis of covariance as a technique for testing the equality of slopes or intercepts in linear regressions (Ludbrook, 2012). Pearson correlation coefficients were therefore used to analyze the significance of differences in the coefficients correlating primary cilia length and function. Asterisks (^*^) denote with statistical significance differences at *p* < 0.05 relative to corresponding control groups as indicated in the figures.

A total of 12 sheep and 12 lambs was used; for each normoxic or hypoxic group, 6 sheep and 6 lambs were used (*N* = 6 sheep and *N* = 6 lambs for normoxia; *N* = 6 sheep and *N* = 6 lambs for hypoxia). From each animal, both kidneys were collected for different studies. Each pair of kidneys were randomly selected for an immediate fixation in formalin or trypsinization for cell culture. From each kidney, a minimum of 10 experimental replicates was sampled. All statistical analysis was done with *GraphPad Prism*, version 5.0b.

## Results

The normoxic control animals had maternal and fetal arterial PO_2_ (PaO_2_) of 102 ± 2 and 25 ± 1 mmHg, respectively. The hypoxic maternal and hypoxic fetal PaO_2_ were 60 ± 2 and 19 ± 1 mmHg, respectively.

### Fetal hypoxic kidneys were characterized by interstitial medullary edema

**Figure 1:**
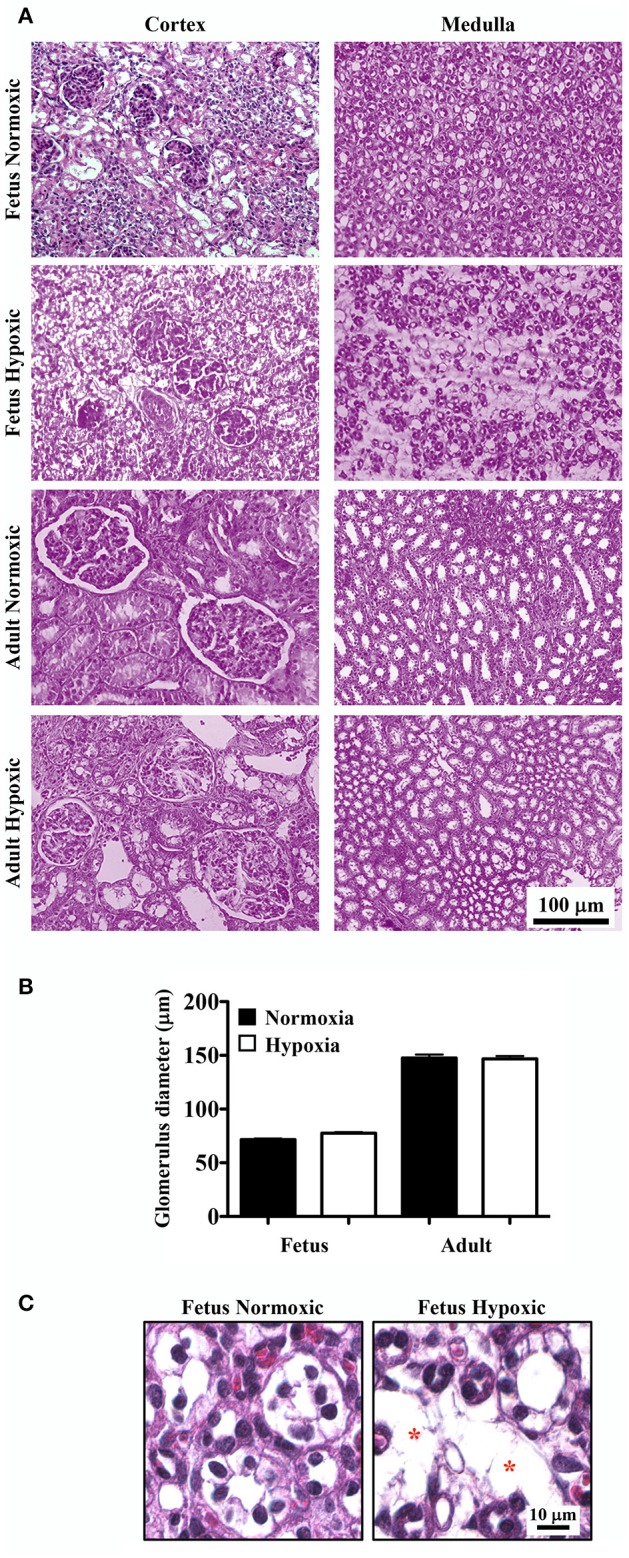
Haemotoxylin and Eosin (H&E) staining to study effects of long-term hypoxia. **(A)** Kidneys were stained with H&E, and representative images were taken at the cortex and medullary regions of the kidneys. **(B)** Glomerular diameters were measured and quantified. **(C)** Representative images show that in fetal hypoxic kidney, tubules are separated by interstitial medullary edema as denoted by asterisks. *N* = 6 animals for each group; statistical analysis was performed with ANOVA followed by the Tukey *post-hoc* test.

Representative images of H&E stained renal tissue sections from normoxic and hypoxic kidneys are presented (Figure 1A). In all kidney sections, light microscopy analysis indicated that the cortex regions of the kidneys had normal glomeruli. There were no significant differences in glomerulus size between normoxic and hypoxic kidneys (Figure 1B). Capillary loops of the glomeruli were also normal, and the number and morphology of endothelial cells were comparable between normoxic and hypoxic tissues.

In normoxic fetal kidneys, all tubules were closely spaced in the interstitium. Tubules were lined with a single layer of epithelia and well-organized nuclei. In hypoxic fetuses, the renal cortex demonstrated some evidence of mesangial and intercapillary cell proliferation. The renal medulla showed apparent atrophic tubules, interstitial fibrosis and edema, and tubular disparition characterized by greater inter-tubular spaces and separation of tubules filled with edema (Figure 1C).

While all tubules in adult sheep were closely spaced in the interstitium and lined by a single layer of cells with well-organized nuclei, some abnormalities were observed in the chronically hypoxic kidney. The hypoxic kidneys showed some proliferation of mesangial cells in the cortex and slight tubular edema in the medullary region. Otherwise, the surrounding tubules appeared normal in both normoxic and hypoxic adult kidneys.

### Hypoxia modulated medullary tubular basement membrane thickness

**Figure 2:**
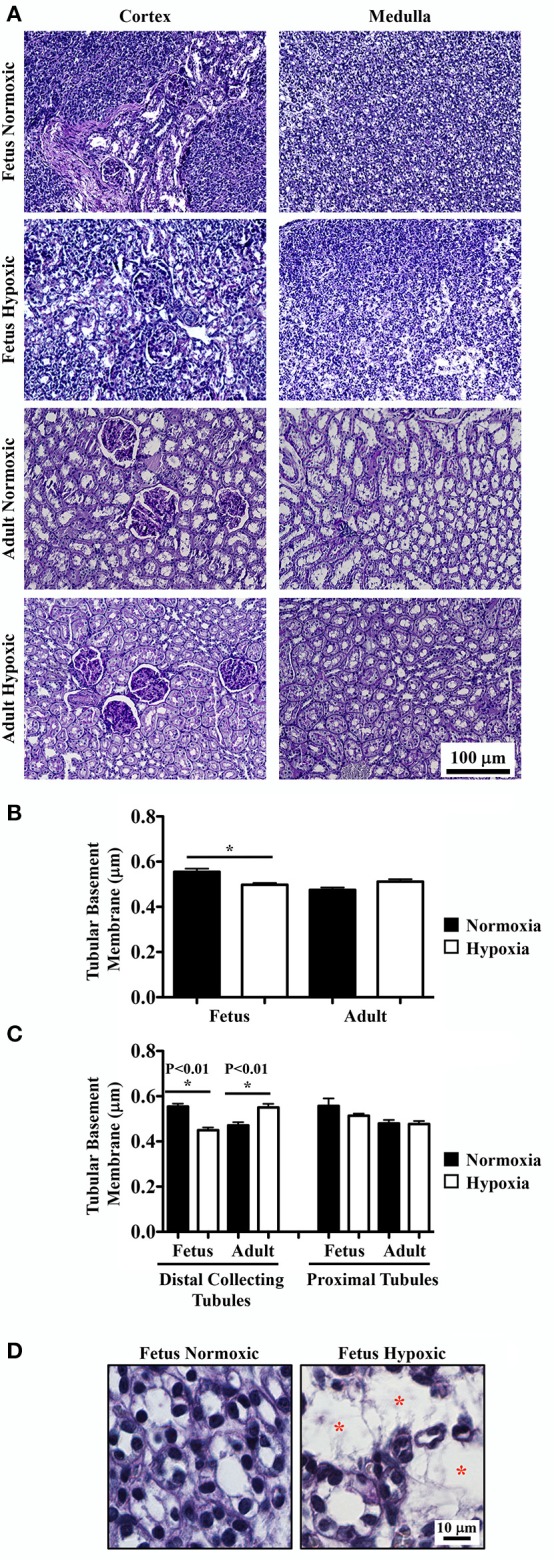
Periodic acid-Schiff (PAS) staining to study effects of long-term hypoxia. **(A)** Kidneys were stained with PAS, and representative images were taken at cortex and medullary regions of the kidneys. **(B)** Tubular basement membrane thickness was measured and quantified. **(C)** More detail analysis of basement membrane was achieved by separation between distal collecting and proximal tubules. **(D)** Representative images show that in fetal hypoxic kidney, tubules were separated by interstitial medullary edema as denoted by red asterisks. ^*^Indicates significant differences between normoxic and hypoxic groups. *N* = 6 animals for each group; statistical analysis was performed with ANOVA followed by the Tukey *post-hoc* test.

PAS staining was done to highlight basement membranes of glomerular capillary loops and tubular epithelia. Renal tissue sections from normoxic and hypoxic kidneys are shown in the representative images (Figure 2A). There were no significant differences in basement membranes thicknesses of glomerular capillary loops between normoxic and hypoxic adult kidneys.

Basement membrane thicknesses of the tubules were significantly less in hypoxic fetal kidneys compared to normoxic fetal kidneys (Figure 2B). Although a reverse trend was observed in adult kidneys, it was not statistically significant in adult normoxic or hypoxic kidneys. To refine the statistical analysis, tubular membrane thicknesses were re-measured by discriminating the tubular origins. In proximal tubules, no significant difference in tubular membrane thickness was observed (Figure 2C). In distal collecting tubules, tubular membranes were significantly thicker in hypoxic than normoxic adult kidneys. As expected, tubular membrane thicknesses were significantly less in hypoxic compared to normoxic fetal kidneys.

As observed in the H&E staining, hypoxic fetal kidneys showed interstitial medullary edema, fibrosis, tubular disparition and atrophy characterized by greater inter-tubular space (Figure 2D).

### Long-term hypoxia induced renal fibrosis

**Figure 3:**
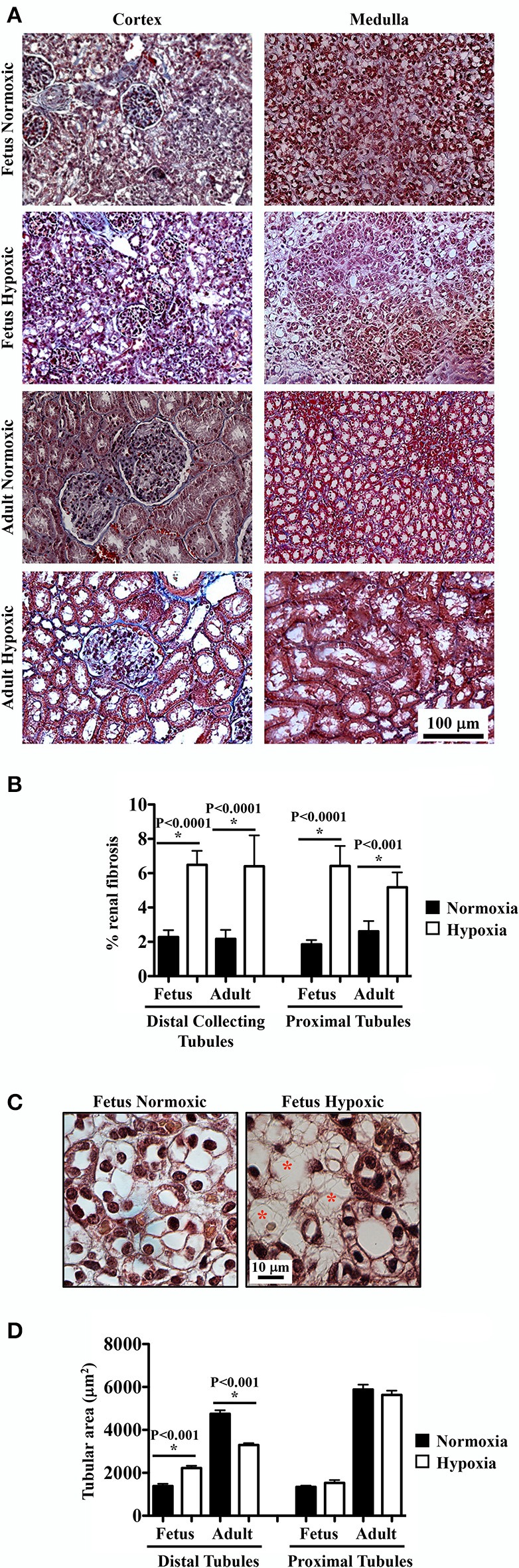
Masson's Trichrome staining to study effects of long-term hypoxia. **(A)** Kidneys were stained with Masson's Trichrome, and representative images were taken at cortex and medullary regions of the kidneys. **(B)** Interstitial renal fibrosis was measured and quantified. **(C)** Representative images show that in fetal hypoxic kidney, tubules were separated by interstitial medullary edema as denoted by red asterisks. **(D)** Tubular areas were measured, quantified and compared. ^*^Indicates significant differences between normoxic and hypoxic groups. *N* = 6 animals for each group; statistical analysis was performed with ANOVA followed by the Tukey *post-hoc* test.

To examine potential fibrosis in the kidneys, Masson's Trichrome staining was performed to highlight deposition of collagen and fibrin fibers. Renal tissue sections from normoxic and hypoxic kidneys are shown in representative images (Figure 3A). The percentage of blue-stained area was calculated using a binary threshold program, which indicated significant fibrosis in hypoxic compared to normoxic kidney tissues (Figure 3B). In normoxic tissues, there was some collagen fibers that were normally distributed around renal tubules. In hypoxic tissues, normal distributions of collagen fibers were observed, but abnormal depositions of collagen/fibrin fibers were also observed throughout tubular epithelia and interstitial spaces.

As observed in the H&E and PAS staining, hypoxic fetal kidney showed interstitial medullary edema characterized by more inter-tubular space (Figure 3C). As also seen in previous staining, hypoxia modulated lumen size of distal tubules in both fetal and adult kidneys (Figure 3D). No significant changes in lumen size were observed in proximal tubules.

### Fetal hypoxic kidney was characterized by longer renal epithelial primary cilia

**Figure 4:**
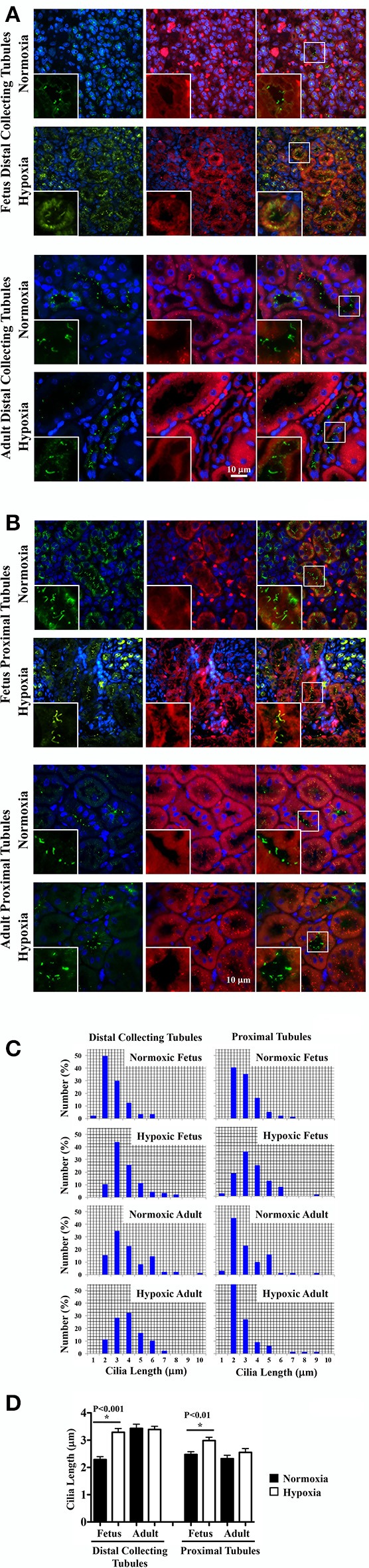
Immunofluorescence staining to study effects of long-term hypoxia on cilia length *in vivo*. **(A)** Kidneys were stained with ciliary marker (acetylated-α-tubulin; green), distal collecting marker (DBA; red) and nucleus marker (DAPI; blue). **(B)** Instead of DBA, kidneys were stained with proximal tubular marker (LTL; red). White boxes show enlargement of the images to depict the magnified view of primary cilia. **(C)** The length of primary cilia was measured and represented in the bar graph to depict length distribution within each group. **(D)** Cilia length was quantified. ^*^Indicates significant differences between normoxic and hypoxic groups. *N* = 6 animals for each group; statistical analysis was performed with ANOVA followed by the Tukey *post-hoc* test.

It remains uncertain if hypoxia can maintain and stabilize the primary cilia or it would inhibit primary cilia formation (Verghese et al., 2011; Ding et al., 2015; Lavagnino et al., 2016; Resnick, 2016). To investigate the effect of chronic hypoxia, primary cilia were labeled with the cilia marker acetylated-α-tubulin. Representative images of renal tissue sections from normoxic and hypoxic kidneys are shown for staining of cilia and tubular markers. Distal collecting (Figure 4A) and proximal (Figure 4B) tubular markers were used to identify cilia length in respective tubules. Cilia measurements were the represented in the bar graphs to compare the distributions of the cilia length (Figure 4C). While hypoxia did not significantly alter cilia length in adult kidneys, fetal kidneys were very susceptible to hypoxia (Figure 4D). Cilia length was significantly longer in hypoxic fetal kidneys than normoxic fetal kidneys.

Of note, that the distal collecting tubules had larger lumen sizes in adult than fetal kidneys. This may be associated with longer cilia in adult than fetal distal collecting tubules. For the first time, our studies show that the length of primary cilia in distal collecting tubules become longer during maturation, while there is no change in proximal tubule cilia length from fetuses to adults.

### Fetal hypoxic kidney had greater sensitivity in response to fluid-shear stress

**Figure 5:**
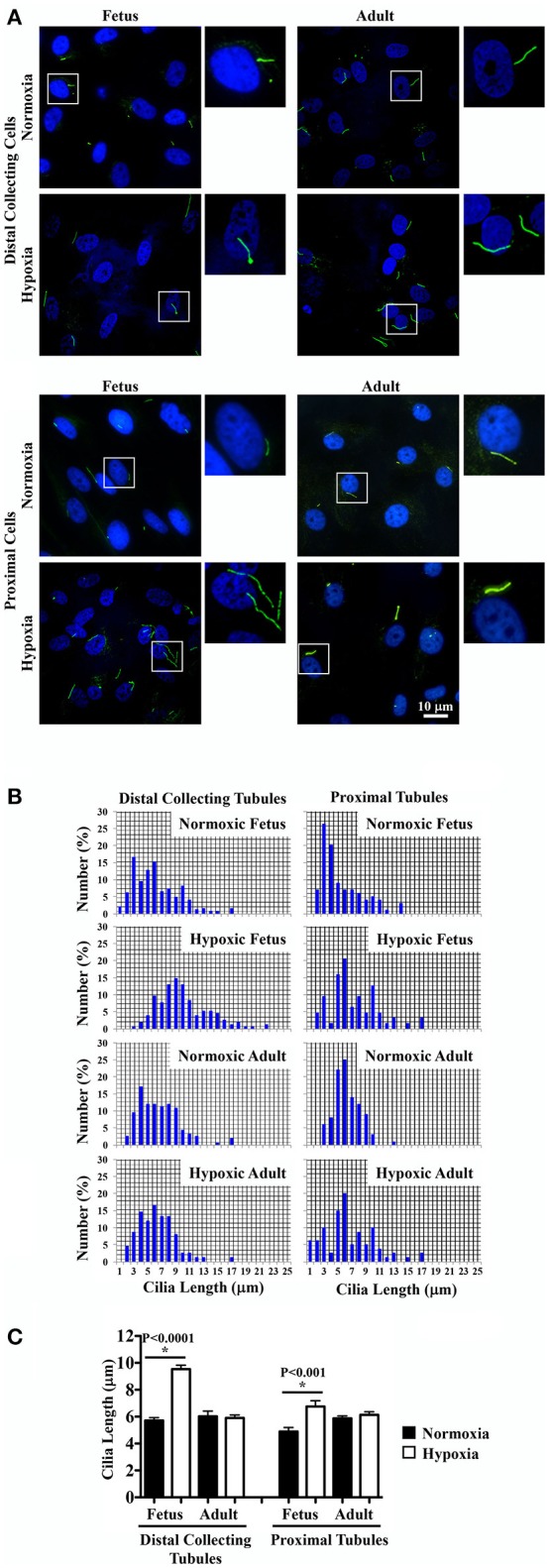
Immunofluorescence staining to study effects of long-term hypoxia on cilia length in *in vitro* cultures. **(A)** Ovine renal epithelia were isolated and stained with ciliary marker (acetylated-α-tubulin; green) and nucleus marker (DAPI; blue). White boxes show enlargement of the images to depict the presence of primary cilia. **(B)** The length of primary cilia was measured and tabulated in the bar graph to depict length distribution within each group. **(C)** Cilia length was quantified. ^*^Indicates significant differences between normoxic and hypoxic groups. *N* = 6 animals for each group; statistical analysis was performed with ANOVA followed by the Tukey *post-hoc* test.

Renal primary cilia are mechanosensory organelles that sense filtrate moving within the tubules. To examine the possibility that hypoxia could alter mechanosensory of cellular responses, renal epithelia were isolated, cultured, stained with a cilia marker and challenged with shear-stress. Representative images of these cells from normoxic and hypoxic kidneys reveal changes in cilia length (Figure 5A). On average about 80% of cells had cilia, and there were no apparent differences in cilia formation between normoxic and hypoxic tissues, or between fetal and adult kidneys. Cilia measurements were then tabulated to compare their distributions (Figure 5B). Cilia measurements were also tabulated to analyze the impact of hypoxia on cilia lengths (Figure 5C). While hypoxia did not significantly alter cilia length in adult cells, cilia length was significantly longer in hypoxic than normoxic fetal cells. Interestingly, trend in cilia length changes was similar for *in vitro* and *in vivo* preparations. When the overall cilia length *in vivo* kidney (Figure 4) or *in vitro* cell culture (Figure 5) were averaged, it was apparent that the *in vitro* cell culture produced much longer cilia than observed *in vivo* (6.35 ± 0.28 μm vs. 2.84 ± 0.12 μm; *p* < 0.00005).

**Figure 6:**
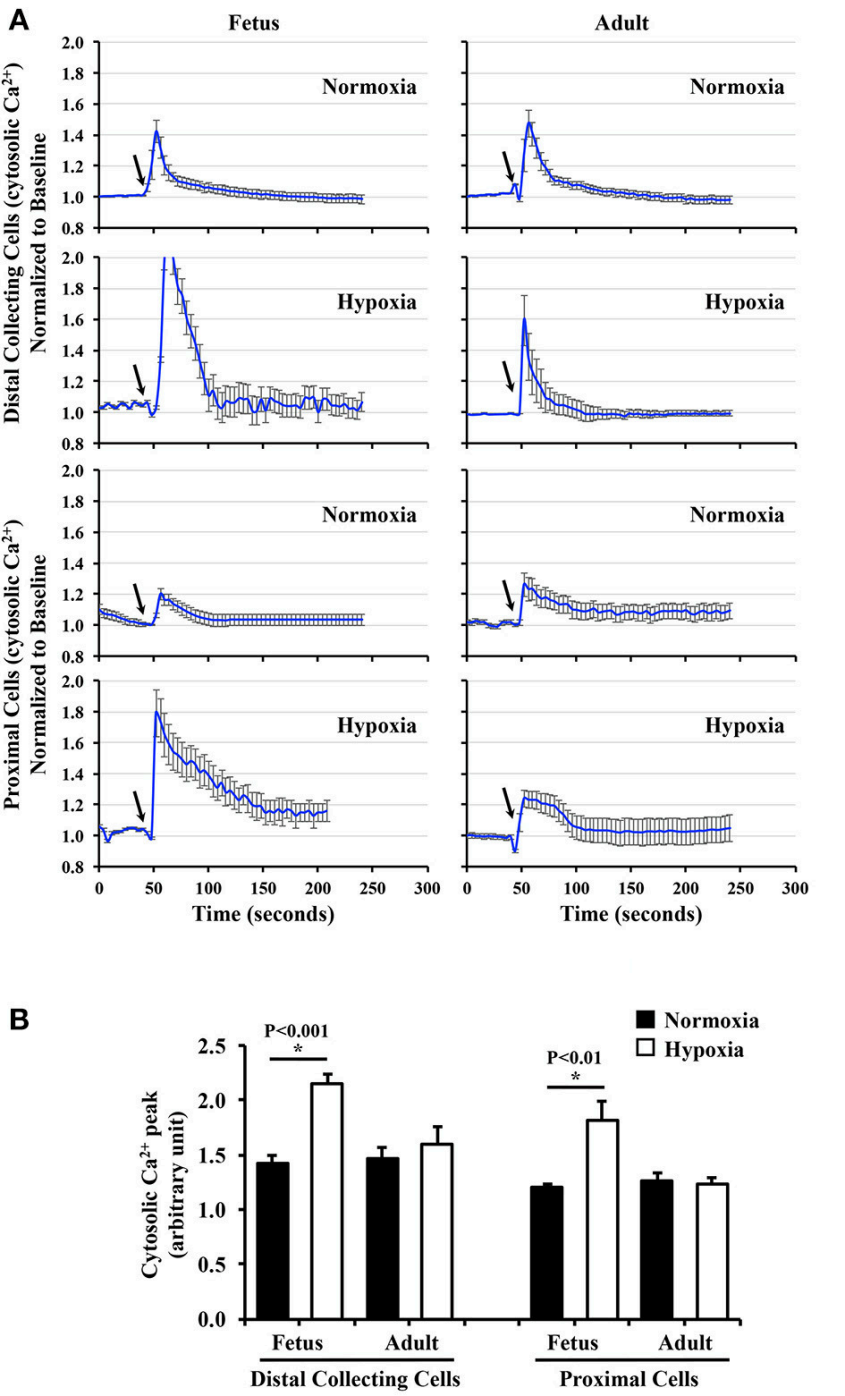
Cytosolic calcium measurement to study effects of long-term hypoxia. **(A)** Cytosolic calcium was measured with Fura-2AM. After equilibration for 30 min, baseline calcium signal was taken for 50 s. Cells were challenged with fluid-shear stress (arrows), and averaged changes in cytosolic calcium for each group were presented in line graphs. **(B)** The peak of calcium changes was selected to depict the function of primary cilia. The bar graph shows the averaged calcium peak in response to fluid-shear stress. *N* = 6 animals for each group; statistical analysis was performed with ANOVA followed by the Tukey *post-hoc* test.

To examine the effect of chronic hypoxia on mechanosensory cilia function, cells were challenged with 0.8 dyne/cm^2^ shear-stress. Changes in cytosolic calcium were averaged and plotted in line graphs (Figure 6A). When peaks of cytosolic calcium were examined, hypoxia significantly enhanced mechanosensory function in fetal epithelial cells (Figure 6B). In contrast, hypoxia did not significantly alter mechanosensory sensitivity in adult cells.

### Hypoxia-induced longer cilia were associated with greater cilia function

**Figure 7:**
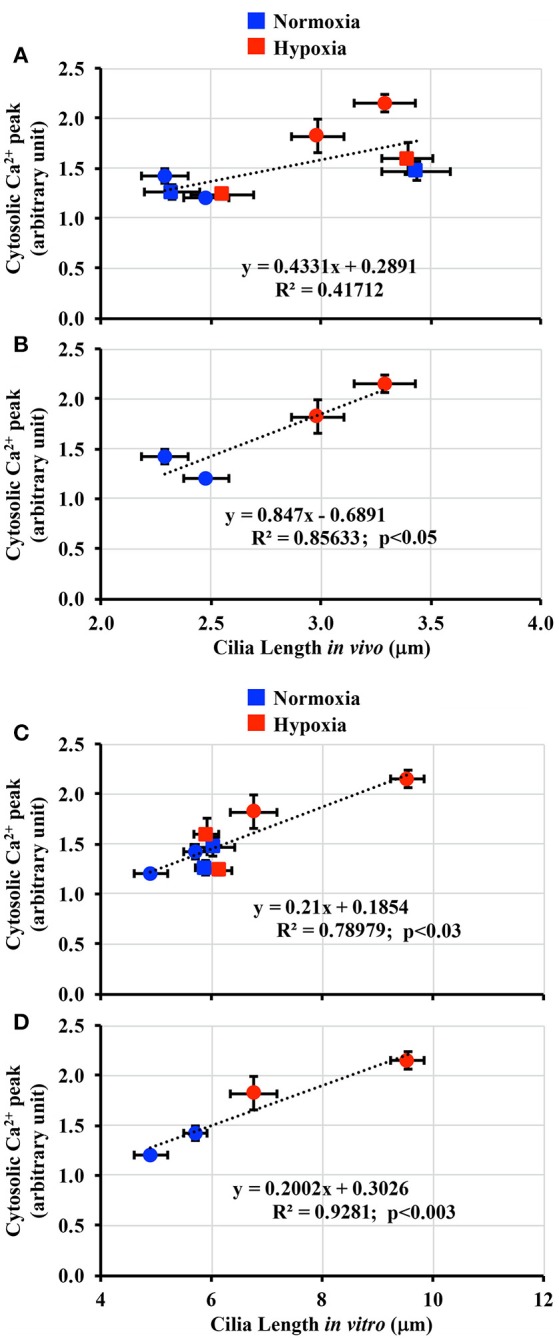
Correlation between cilia length-function. **(A)** Cilia length-function relation was plotted in a dot plot from both fetal and adult groups *in vivo*. **(B)** Because hypoxia affected mainly on fetal renal cilia, the length-function relation showed a greater correlation when plotted only for fetus. **(C)** Cilia length was next taken from the *in vitro* measurement. Cilia length-function relation was plotted in a dot plot from both fetal and adult groups. **(D)** Because hypoxia affected mainly on fetal renal cilia, the length-function relation showed a greater correlation when plotted only for fetus. *N* = 6 animals for each group; statistical analysis was performed with the Pearson correlation coefficient test.

It has been hypothesized that longer cilia are more sensitive to fluid-shear stress (Abdul-Majeed et al., 2012; Upadhyay et al., 2014). To examine the effect of hypoxia on cilia length-function relationship, both *in vivo* (Figure 4) and *in vitro* (Figure 5) cilia lengths were used to assess changes in cilia function. When *in vivo* cilia length was plotted against cilia function, no apparent length-function association was observed (Figure 7A; *R*^2^ = 0.42). Because hypoxia did not alter cilia length in adult kidneys, length-function relationship was next analyzed only in fetal kidneys. This revealed significant correlation within length-function relationships (Figure 7B; *R*^2^ = 0.86). When *in vitro* cilia length was analyzed, the cilia function was significantly correlated with cilia length following either inclusion (Figure 7C; *R*^2^ = 0.79) or exclusion (Figure 7D; *R*^2^ = 0.93) of adult kidneys.

## Discussion

Although kidneys are important for regulation of body fluid, pH, electrolyte, hormone and overall metabolism, the effects of chronic hypobaric hypoxia on fetal kidneys have never been examined. We therefore study the effects of chronic hypoxia on fetal and adult sheep kidneys within the cortex and medullary regions. Effects of hypoxia on mechanosensory primary cilia are also evaluated. Our studies suggest that: (1) there are no significant differences in glomerulus size between normoxic and hypoxic kidneys; (2) the hypoxic kidneys show proliferation of mesangial cells, medullary edema and fibrosis; (3) hypoxia modulates changes in tubular basement membranes; and (4) compared to adult kidneys, fetal kidneys are more susceptible to hypoxia-induced tubular disparition and atrophy, characterized by alterations in primary cilia and function.

A previous study has demonstrated enlargement of renal glomeruli in hypoxic children (Naeye, 1965). However, our studies do not support glomerulus enlargement in the hypoxic fetal or adult sheep kidneys. This discrepancy could potentially be due to an age-specific effect. For example, glomerular enlargement only occurs after the first month of life, while the size of glomeruli is normal at birth in those hypoxic children (Naeye, 1965). Since all of these children die as a result of unknown illness, it is also possible that other genetic and environmental factors contributed to the glomerulus enlargement. For example, an oxonic acid diet in rats is known to cause hyperuricemia through elevated plasma renin activity (Eraranta et al., 2008). Glomerular hypertrophy is observed in oxonic acid-induced hyperuricemia and can be prevented by ACE inhibitor therapy in the rats (Nakagawa et al., 2003).

Abnormal mesangial cells, edema and fibrosis are observed in our hypoxic kidneys. This is possibly a result from interstitial renal injury due to chronic hypobaric hypoxia. Of note, the deposition of collagen and fibrin fiber that functions as a by-product of a reparative process, is commonly used as an index of various renal injury (Ma et al., 1998; Kaissling and Le Hir, 2008; Forbes et al., 2012). Although hypoxia is one of the stimuli that drives chronic kidney disease (Fu et al., 2016), our studies further reveal that hypoxia could alter the thickness of tubular basement membranes. Interestingly, hypoxia only induces changes in basement membranes of distal collecting tubules. This could be due to medullary regions that operate within a relatively lower range of pO_2_, and it is therefore more susceptible to hypoxic injury than proximal regions (Heyman et al., 1997; El Sabbahy and Vaidya, 2011). Greater susceptibility of medullary regions to hypoxia might thus contribute to an increase in collecting distal tubular basement membrane of adult kidneys. Unlike adult medullary tissues, however, hypoxia actually causes a decrease in medullary basement membrane in fetal kidney. This could possibly be due to tubular disparition and atrophy, which are very apparent in the fetal medulla. The thinning in basement membrane in medullary tissues might therefore be associated with a degeneration of tubular structure, as observed in fetal hypoxic kidneys.

Based on the histological analyses, fetal kidneys are more susceptible to hypoxia, possibly due to a “triple-hit hypoxia” phenomenon. In addition to the hypobaric hypoxia in the atmosphere, fetal kidneys are impacted by three additional factors (triple-hit). First, chronic hypoxia induces vasoconstriction in sheep uterine arteries during gestation (Hu et al., 2012; Xiao et al., 2013). This maladaptation of the uteroplacental circulation can result in reduced tissue perfusion and further exacerbate the effects of hypoxia. Second, in hypoxic fetal sheep there is a rapid drop in fetal heart rate and a rise in mean arterial blood pressure that redistributes blood flow to the heart and brain at the expense of the renal circulation. Hypoxia therefore reduces renal blood flow, resulting in renal hypoperfusion (Robillard et al., 1986; Green et al., 1997). Third, the unique fetal circulation system allows oxygen-rich blood from the aorta to mix with oxygen-poor blood before reaching renal circulation (Nakamura et al., 1987). Thus, the fetal circulation system reduces oxygenation support to the kidneys.

Our results also indicate that greater susceptibility to hypoxia in fetal kidneys could be associated with changes in primary cilia length and function throughout the nephrons. Primary cilia are sensory organelles rising from the apical surface of most mammalian cells. Cilia are activated during bending by fluid-flow, which in turn initiates an intracellular calcium response (Praetorius and Spring, 2001; Liu et al., 2005; Nauli et al., 2013). Previous studies demonstrate a link between cilia length and hypoxia-inducible mechanisms. In tendon cells, hypoxia inhibits primary cilia formation and cellular mechano-responsiveness (Lavagnino et al., 2016). However, studies in different hypoxic models using renal epithelial cells indicate that primary cilia are longer and that expression of HIF maintains primary cilia length (Verghese et al., 2011; Ding et al., 2015). Interestingly, the renal epithelial cilia become more flexible during hypoxia (Resnick, 2016), and it thus might alter cilia function. Consistent with this view, a correlation between cilia length and function in response to chronic hypoxia is observed in our studies. Interestingly, greater cilia length in hypoxic fetal kidney *in vivo* are maintained as measured in *in vitro* cell culture. This could be due to epigenetic changes occurring during hypoxia. Of note, long-term hypoxia during gestation has been reported to cause epigenetic adaptation in the sheep (Dasgupta et al., 2012; Chen et al., 2014).

**Figure 8:**
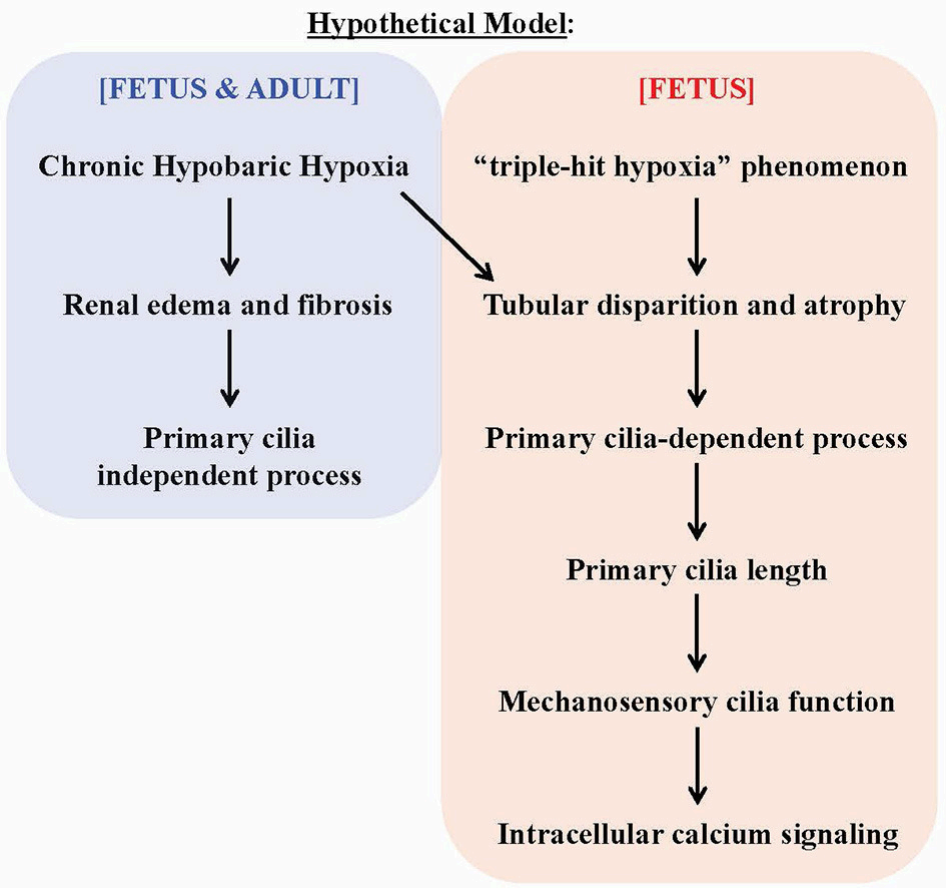
Hypothetical model on the effects of chronic hypoxia-induced renal injury on primary cilia. The chronic hypobaric hypoxia induces kidney injuries, such (as) renal edema and fibrosis. Such injuries may be considered “mild” that does not alter primary cilia length and function. The triple-hit hypoxia, compounding the chronic hypoxia, is a phenomenon independently observed in fetus. The tripe-hit hypoxia is characterized by 1) chronic hypoxia-induced maladaptation of uteroplacental vasoconstriction, 2) redistribution of blood flow away from the fetal kidneys and 3) the unique oxygen-poor blood circulation in the fetal kidneys. This triggers cilia length increase, which in turn improves cilia function. The overall response will result in changes of intracellular calcium signaling.

Our studies indicate that chronic hypobaric hypoxia could induce renal injury (Figure 8). Renal hypoperfusion as a result from the “triple-hit hypoxia” phenomenon seen in fetus would further induce tubular disparition and atrophy, a process that modulated changes in cilia length and function. This, in turn, induces an increase in intracellular calcium fluxes in response to fluid-shear stress. Our results potentially point to a complex renal injury caused by chronic hypoxia with regards to primary cilia.

It has been reported that kidney compensation induced by a reduction of renal mass results in primary cilia elongation, and this elongation is associated with an increased production of reactive oxygen species (Han et al., 2016). In addition, renal primary cilia lengthens after acute tubular necrosis (Verghese et al., 2009). The contribution of primary cilia from acute to chronic injury is also confirmed when a ciliary protein is inactivated. In this case, renal damages are more pronounced following ischemia/reperfusion, and this induces microcyst formation (Bastos et al., 2009). In addition to the association of human renal carcinoma and primary cilia (Ding et al., 2015), acute injury can induce chronic kidney disease through cyst formation in the absence of normal renal cilia (Patel et al., 2008). Furthermore, mechanosensory function of cilia is abnormal in chronic kidney disease patients (Nauli et al., 2006; Xu et al., 2007). Based on these findings, we speculate that the increases in hypoxia-induced cilia-related calcium signaling in hypoxic fetal kidneys serves a potential mechanism associated with renal injury. Without doubt, future studies on the hypoxia-induced changes in intracellular calcium are warranted.

## Author contributions

KS analyzed data, contributed to drafting the manuscript and oversaw the entire progress. JC and RS performed the calcium studies. JS and RP performed cilia measurements and kidney staining. KA assisted in sample collections. WP and LZ provided the kidney samples. LZ and SMN finalized the manuscript and oversaw the research project.

### Conflict of interest statement

The authors declare that the research was conducted in the absence of any commercial or financial relationships that could be construed as a potential conflict of interest.
